# Full Genomic Characterization of a Saffold Virus Isolated in Peru

**DOI:** 10.3390/pathogens4040816

**Published:** 2015-11-20

**Authors:** Mariana Leguia, Steev Loyola, Jane Rios, Diana Juarez, Carolina Guevara, Maria Silva, Karla Prieto, Michael Wiley, Matthew R. Kasper, Gustavo Palacios, Daniel G. Bausch

**Affiliations:** 1 U.S. Naval Medical Research Unit No. 6 (NAMRU-6), Callao 2, Peru; E-Mails: steev.loyola@med.navy.mil (S.L.); jane.s.rios.fn@mail.mil (J.R.); diana.s.juarez2.fn@mail.mil (D.J.); carolina.guevara.fn@mail.mil (C.G.); maria.e.silva19.fn@mail.mil (M.S.); matthew.r.kasper2.mil@mail.mil (M.R.K.); 2 Center for Genome Sciences, United States Army Medical Research Institute for Infectious Diseases (USAMRIID), Frederick, MD 21702, USA; E-Mails: karla.prieto.ctr@mail.mil (K.P.); michael.r.wiley19.ctr@mail.mil (M.W.); gustavo.f.palacios.ctr@mail.mil (G.P.); 3 U.S. Naval Medical Research Unit No. 6 (NAMRU-6), Callao 2, Peru; Tulane School of Public Health and Tropical Medicine, New Orleans, LA 70112, USA; E-Mail: bauschd@who.int

**Keywords:** Saffold virus, *Cardiovirus*, full genome, Peru

## Abstract

While studying respiratory infections of unknown etiology we detected Saffold virus in an oropharyngeal swab collected from a two-year-old female suffering from diarrhea and respiratory illness. The full viral genome recovered by deep sequencing showed 98% identity to a previously described Saffold strain isolated in Japan. Phylogenetic analysis confirmed the Peruvian Saffold strain belongs to genotype 3 and is most closely related to strains that have circulated in Asia. This is the first documented case report of Saffold virus in Peru and the only complete genomic characterization of a Saffold-3 isolate from the Americas.

## 1. Introduction

Saffold virus (SAFV) was first described in 2007, after it was isolated from a stool sample taken in San Diego, in 1981, from an eight-month-old female with fever of unknown origin [1]. SAFV belongs to the *Cardiovirus* genus of the *Picornaviridae* family. The *Cardiovirus* genus is composed of two species, encephalomyocarditis virus, for which only one serotype has been reported, and Theilovirus, for which 14 distinct serotypes are known: SAFV 1–11, Theiler’s murine encephalomyelitis virus, Thera virus, and Vilyuisk human encephalomyelitis virus [2]. SAFVs are ubiquitous in populations around the world and have been linked to respiratory and gastrointestinal infections early in life [3,4,5,6]. Despite their extensive distribution, only a handful of SAFV genomic sequences from the Americas have been described [7,8,9]. Furthermore, many of the sequences publicly available are not complete genomes, but rather very small fragments of partial VP1 sequences. Aside from its original 1981 isolation in the USA, SAFV-1 has only been reported in Bolivia [9]. Other serotypes, including SAFV-2, 4, and 9, have been reported in Bolivia, Brazil, Canada, and the USA [7,8,9]. For the remaining serotypes, including SAFV-3, 5, 6, 7, 8, 10, and 11, there are no known reports of isolates from the Americas. Here, we provide complete genomic characterization of a SAFV-3 isolate collected from a two-year-old female from the Amazonian area of Maynas, in Loreto, Peru.

## 2. Results and Discussion

**Figure 1:**
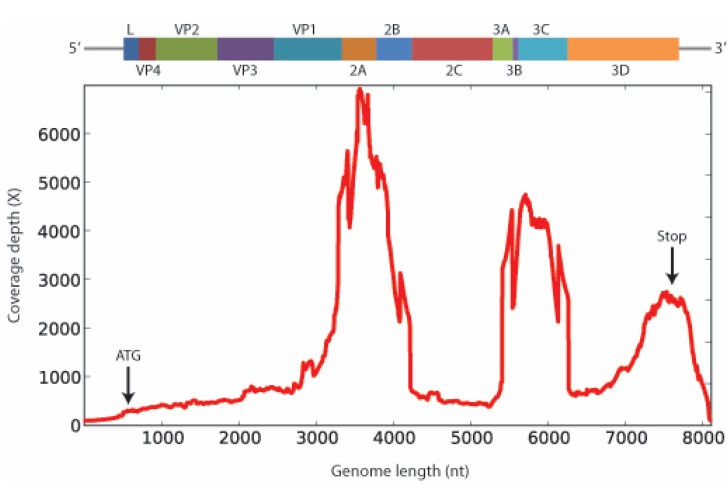
Schematic representation of the genomic organization of the SAFV polyprotein, including all genes (L, VP1, VP2, VP3, VP4, 2A, 2B, 2C, 3A, 3B, 3C, and 3D) drawn to approximate scale (top), followed by the corresponding coverage depth by Illumina sequencing (bottom).

The SAFV strain described here was isolated from an oropharyngeal swab collected from a two-year-old female who presented with diarrhea, heart murmur, and symptoms of respiratory illness, including headache, sore throat, cough, rhinorrea, and dyspnea. The patient was identified during routine respiratory surveillance efforts carried out by investigators from the U.S. Naval Medical Research Unit No.6 in Peru and neighboring countries, with institutional review board approval from all implicated partners (Protocols NMRCD.2010.0010 and NMRCD.2010.0008). The sample was initially cultured in LLC-MK2, MDCK, and Vero-E6 cells, but by day 18 cytopathic effect (CPE) had only been observed in LLC-MK2 cells and no pathogens had been identified in the original sample using traditional PCR or ELISA-based approaches. Additional analyses of the CPE-positive LLC-MK2 culture supernatant, using a highly multiplexed MassTag PCR approach that can detect over 20 respiratory pathogens simultaneously (Influenza viruses A and B; respiratory syncytial viruses A and B; human parainfluenza viruses 1–4; human metapneumovirus; coronavirus OC43 and 229E; enterovirus; rhinovirus; adenovirus; *Chlamydia pneumoniae*; *Legionella pneumophila*; *Mycoplasma pneumoniae*; *Streptococcus pneumoniae*; *Neisseria meningitidis*; *Haemophilus influenzae*; *Mycobacterium* *tuberculosis*; *Moraxella catarrhalis*; and *Bordetella pertussis*) [10], also failed to identify any potential etiology. The sample then entered a pathogen discovery pipeline based on unbiased next-generation sequencing that eventually produced a match to a Japanese isolate of SAFV-3 (Accession #HQ902242.1) with 98% identity at the nt level. The consensus sequence generated (Figure 1) has been named SAFV-3-Maynas-Loreto-Peru-2012 (Accession #KP972594) based on the location and year of sample collection. The contig is 8064 nt in length and contains a complete open reading frame (ORF) of 2296 aa.

**Figure 2:**
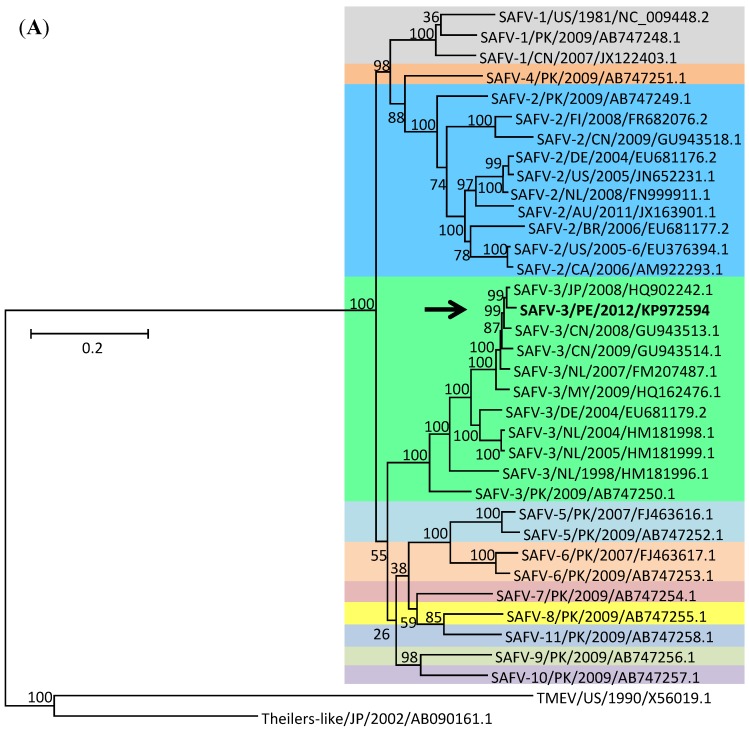

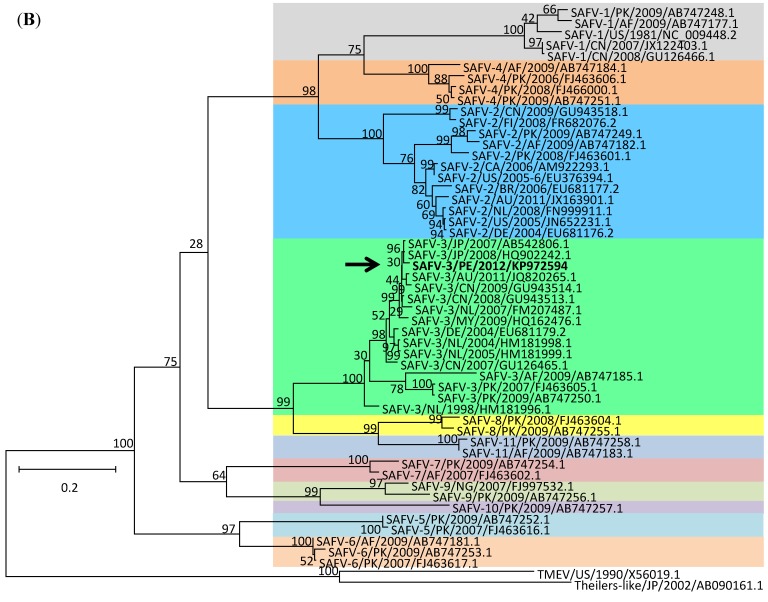
Phylogenetic analyses of SAFV full genome (**A**) and complete VP1 (**B**) sequences. Each strain is labeled using standard identifiers, including virus type, country of isolation (using ISO country codes), isolate name (if any), year of isolation, and GenBank accession number. Additionally, SAFV1-11 serotypes are color-coded for easy viewing. The Peruvian isolate described here is highlighted in bold and with an arrow. Full genome trees were constructed using a total of 36 publicly available complete genome sequences covering serotypes 1–11. Full VP1 trees were constructed using those same sequences plus 17 additional sequences containing complete VP1 genes. Scale bars represent the number of substitutions per site.

To further characterize our strain, we conducted phylogenetic analyses of both the full genome (ORF only, 6888 nt, 2296 aa) and the complete viral protein 1 (VP1) (810 nt, 270 aa). This latter region is expected to be under positive selection in order to avoid recognition by the host’s immune system, and thus could potentially return different phylogenetic results when compared to the whole genome. To maintain sampling diversity as large as possible, trees were constructed using publicly available reference sequences that represent the totality of the diversity of SAFV strains in terms of genotype, year of isolation, and geographical origin (Table 1). The full genome tree includes 34 complete genome sequences covering serotypes 1–11, whereas the full VP1 tree includes the same 34 sequences used for the full genome tree plus 7 additional available sequences containing complete VP1 genes. Phylogenetic analyses of both full genome and complete VP1 sequences confirm that the Peruvian SAFV strain collected in 2012 belongs to genotype 3 and is most closely related to Asian strains (Figure 2). We also constructed an additional VP1 tree, this time containing partial VP1 sequences available from several Bolivian isolates (Figure S1). Although there are minor differences in the branching patterns of the two VP1 trees, the Peruvian strain remains closely associated with Asian strains within the SAFV-3 group despite the fact that a number of additional South American SAFV strains were included in the analysis. The fact that the Peruvian isolate is most closely related to SAFV strains that have circulated in Asia rather than in Europe, for example, should not be surprising given the available information. Specifically, there are no other reports of SAFV-3 from any country in the Americas, indicating that information from additional American strains will be needed to establish more robust phylogenetic relationships in support of theories of SAFV movements throughout the world. As it stands, the information presented here can only be used to support grouping of the Peruvian SAFV strain into group 3, with particular similarity to Asian strains. Nevertheless, it is worth noting that Peru has historically received a large number of immigrants from Asia, particularly from China and Japan. Along with further characterization of additional strains, this observation could be used to support a theory of introduction of SAFV into the Americas directly from Asia.

## 3. Experimental Section

### 3.1. Cell Culture

Oropharyngeal swabs were inoculated into Rhesus monkey kidney (LLC-MK2), Madin-Darby canine kidney (MDCK), and African Green monkey kidney (Vero-E6) cells grown in E-MEM (Quality Biological, 112-018-101. Quality Biological Inc.: Gaithersburg, MD, USA) supplemented with 10% fetal bovine serum (Sigma, F4135. Sigma-Aldrich Co.: St. Louis, MO, USA), 1× sodium pyruvate (Quality Biological, 116-079-721), 1× antibiotic/antimycotic solution (Sigma, A5955), and 100U/mL penicillin-streptomycin (Gibco, 15140-122. Thermo Fisher Scientific: Waltham, MA, USA). Cultures were maintained at 37 °C and 5% CO_2_ until was observed.

### 3.2. MassTag PCR

CPE-positive culture supernatants were extracted using the QIAamp cador pathogen mini kit (QIAGEN, 54104. QIAGEN: Valencia, CA, USA) according to the manufacturer’s instructions and nucleic acids were analyzed by MassTag PCR essentially as described [10,11]. Briefly, RNAs were reverse-transcribed using SuperScript II (Thermo Fisher, 18064-014) and random primers (Thermo Fisher, 48190-011). Reverse transcription products were amplified using a panel of primers labeled with photocleavable mass codes (Agilent, custom. Agilent Technologies: Santa Clara, CA, USA) targeting Influenza viruses A and B, respiratory syncytial viruses A and B, human parainfluenza viruses 1–4, human metapneumovirus; coronavirus OC43 and 229E, enterovirus, rhinovirus, and adenovirus. Upon removal of unincorporated primers, tags were released by UV irradiation and analyzed using a 6100 Series Single Quadrupole LC/MS System (Agilent Technologies).

### 3.3. Sequencing Library Preparation

CPE-positive culture supernatants were preserved in TRIzol LS (Thermo Fisher, 10296-028) and RNA was extracted using Direct-zol™ RNA MiniPrep kit (Zymo Research, R2050) according to the manufacturer’s instructions. RNAs were converted to cDNA and amplified using sequence-independent single primer amplification as described [12] with the following modifications. To enhance coverage of the terminal ends, an oligo containing three rGTP at the 3′ end (GCCGGAGCTCTGCAGATATCGGCCATTATGGCCrGrGrG) and the FR40RV-T primer [12] were added during first-strand synthesis and the reverse transcriptase was changed to Maxima H Minus (Thermo Fisher, EP0751), which has terminal transferase activity that enables addition of the rGTP containing oligo to the 5′ end during cDNA synthesis. Amplicons were sheared to ~400 bp and used as starting material for TRUseq libraries (Illumina FC-121-4001. Illumina, Inc.: Hayward, CA, USA) prepared according to the manufacturer’s instructions.

### 3.4. Sequencing and Bioinformatics

Sequencing was performed on a MiSeq using a 300 cycle kit (Illumina MS-102-2002). Cutadapt [13] and Prinseq-lite [14] were used to trim primers and remove poor quality reads, respectively. Reads were assembled into contigs using Ray Meta [15] and annotation was done by BLAST in combination with custom scripts.

### 3.5. Phylogenetics

A number of available SAFV sequences were downloaded from GenBank to serve as references (Table 1). To cover as many genotypes, years, and geographical regions as possible, we considered 34 complete genomes (at least 6888 nt covering all 2296 aa of representative SAFV1-11 isolates, including the Peruvian isolate), 17 complete VP1 sequences (810 nt covering all 270 aa), and seven additional partial VP1 sequences (at least 312 nt) specifically from the Americas. Theiler’s murine encephalomyelitis virus and Theilers-like virus genomes were used as outgroups in the analysis. Alignments were constructed using Muscle and trees were generated using the Maximum Likelihood algorithm with 2000 bootstrap replicates in Mega 6.0 [16,17]. Genetic distances were calculated using a General Time Reversible Gamma Distributed model.

**Table 1:** Sequences used for phylogenetic analysis. The Peruvian isolate is highlighted in gray. Sequences from isolates with complete genomes were used for both full genome and VP1 phylogenetic trees. Sequences from isolates with partial VP1 sequences were used for an additional VP1 tree that is almost identical to the complete VP1 tree (Figure S1). Abbreviations: SAFV-Saffold virus; TMEV-Theiler’s murine encephalomyelitis virus.

Virus	Accession #	Year	Location	Sequence Type	Length (nt)
SAFV-1	NC_009448.2	1981	USA	Complete genome	8115
SAFV-1	JX219488.1	2002	Bolivia	Partial VP1	321
SAFV-1	JX219490.1	2003	Bolivia	Partial VP1	356
SAFV-1	JX122403.1	2007	China	Complete genome	7856
SAFV-1	GU126466.1	2008	China	Complete VP1	822
SAFV-1	AB747177.1	2009	Afghanistan	Complete VP1	847
SAFV-1	AB747248.1	2009	Pakistan	Complete genome	8078
SAFV-2	JX219484.1	2002	Bolivia	Partial VP1	357
SAFV-2	JX219485.1	2003	Bolivia	Partial VP1	376
SAFV-2	EU681176.2	2004	Germany	Complete genome	7842
SAFV-2	JN652231.1	2005	USA	Complete genome	7885
SAFV-2	EU376394.1	2005–2006	USA	Complete genome	7961
SAFV-2	EU681177.2	2006	Brazil	Complete genome	7809
SAFV-2	AM922293	2006	Canada	Complete genome	6882
SAFV-2	FR682076.2	2008	Finland	Complete genome	8046
SAFV-2	FN999911.1	2008	Netherlands	Complete genome	8045
SAFV-2	FJ463601.1	2008	Pakistan	Complete VP1	816
SAFV-2	AB747182.1	2009	Afghanistan	Complete VP1	856
SAFV-2	GU943518.1	2009	China	Complete genome	7846
SAFV-2	AB747249.1	2009	Pakistan	Complete genome	8075
SAFV-2	JX163901.1	2011	Australia	Complete genome	7781
SAFV-3	HM181996.1	1998	Netherlands	Complete genome	7984
SAFV-3	EU681179.2	2004	Germany	Complete genome	7846
SAFV-3	HM181998.1	2004	Netherlands	Complete genome	7991
SAFV-3	HM181999.1	2005	Netherlands	Complete genome	7991
SAFV-3	GU126465.1	2007	China	Complete VP1	810
SAFV-3	AB542806.1	2007	Japan	Complete VP1	813
SAFV-3	FM207487.1	2007	Netherlands	Complete genome	8051
SAFV-3	FJ463605.1	2007	Pakistan	Complete VP1	810
SAFV-3	GU943513.1	2008	China	Complete genome	8054
SAFV-3	HQ902242.1	2008	Japan	Complete genome	8082
SAFV-3	AB747185.1	2009	Afghanistan	Complete VP1	835
SAFV-3	GU943514.1	2009	China	Complete genome	7853
SAFV-3	HQ162476.1	2009	Malaysia	Complete genome	8073
SAFV-3	AB747250.1	2009	Pakistan	Complete genome	8079
SAFV-3	JQ820265.1	2011	Australia	Complete VP1	843
SAFV-3	KP972594	2012	Peru	Complete genome	8064
SAFV-4	JX219486.1	2002	Bolivia	Partial VP1	315
SAFV-4	JX219487.1	2003	Bolivia	Partial VP1	312
SAFV-4	FJ463606.1	2006	Pakistan	Complete VP1	813
SAFV-4	FJ463600.1	2008	Pakistan	Complete VP1	813
SAFV-4	AB747184.1	2009	Afghanistan	Complete VP1	845
SAFV-4	AB747251.1	2009	Pakistan	Complete genome	8027
SAFV-5	FJ463616.1	2007	Pakistan	Complete genome	7639
SAFV-5	AB747252.1	2009	Pakistan	Complete genome	8083
SAFV-6	FJ463617.1	2007	Pakistan	Complete genome	7410
SAFV-6	AB747181.1	2009	Afghanistan	Complete VP1	822
SAFV-6	AB747253.1	2009	Pakistan	Complete genome	8084
SAFV-7	FJ463602.1	2007	Afghanistan	Complete VP1	816
SAFV-7	AB747254.1	2009	Pakistan	Complete genome	8087
SAFV-8	FJ463604.1	2008	Pakistan	Complete VP1	810
SAFV-8	AB747255.1	2009	Pakistan	Complete genome	8081
SAFV-9	JX219494.1	2002	Bolivia	Partial VP1	343
SAFV-9	FJ997532.1	2007	Nigeria	Complete VP1	789
SAFV-9	AB747256.1	2009	Pakistan	Complete genome	8081
SAFV-10	AB747257.1	2009	Pakistan	Complete genome	8083
SAFV-11	AB747183.1	2009	Afghanistan	Complete VP1	829
SAFV-11	AB747258.1	2009	Pakistan	Complete genome	8079
TMEV	X56019.1	1990	USA	Complete genome	8101
Theilers-like virus	AB090161.1	2002	Japan	Complete genome	8021

## 4. Conclusions

Our case report constitutes the first isolation of SAFV in Peru and the only complete genomic characterization of a SAFV-3 isolate from the Americas. Although the characteristics of the patient from whom the specimen was isolated agree with previous reports that the virus affects young children and that it is linked to both respiratory and gastrointestinal infections, little is known about the prevalence of SAFV infections in South American populations. At least two serological studies in Peru have shown the presence of neutralizing antibodies to closely related viruses of the *Cardiovirus*genus [18,19]. One of these studies reported that 21% of the population of the Amazonian city of Iquitos had neutralizing antibodies to encephalomyocarditis virus [18]. Interestingly, the authors also reported elevated cross-reactivity rates (43.5%), suggesting that many sero-conversions could be due to the presence of closely related members of the *Cardiovirus* genus, including SAFV. Upcoming studies looking specifically at SAFVs should help elucidate both the prevalence and virulence of these pathogens, particularly in vulnerable populations. In turn, these may allow more robust characterizations of SAFV evolution in the Americas.

## Supplementary Files

Supplementary File 1

## Disclaimers

The views expressed in this article are those of the authors and do not reflect the official policy or position of the Department of the Navy, Department of Defense, or the U.S. Government. We are military service members or employees of the U.S. Government. The work was prepared as part of our official duties. Title 17 U.S.C. §105 provides that “Copyright protection under this title is not available for any work of the United States Government.” Title 17 U.S.C. §101 defines U.S. Government work as a work prepared by military service members or employees of the U.S. Government as part of that person’s official duties. The work was supported by work unit number 847705.82000.25GB.B0016. The study protocol was approved by NAMRU-6’s IRB in compliance with all applicable Federal regulations governing the protection of human subjects.

## References

[1] Jones M.S., Lukashov V.V., Ganac R.D., Schnurr D.P. (2007). Discovery of a novel human picornavirus in a stool sample from a pediatric patient presenting with fever of unknown origin. J. Clin. Microbiol..

[2] Carocci M., Bakkali-Kassimi L. (2012). The encephalomyocarditis virus. Virulence.

[3] Naeem A., Hosomi T., Nishimura Y., Alam M.M., Oka T., Zaidi S.S., Shimizu H. (2014). Genetic diversity of circulating saffold viruses in Pakistan and Afghanistan. J. Gen. Virol..

[4] Taboada B., Espinoza M.A., Isa P., Aponte F.E., Arias-Ortiz M.A., Monge-Martinez J., Rodriguez-Vazquez R., Diaz-Hernandez F., Zarate-Vidal F., Wong-Chew R.M. (2014). Is there still room for novel viral pathogens in pediatric respiratory tract infections?. PLoS ONE.

[5] Yodmeeklin A., Khamrin P., Chuchaona W., Saikruang W., Malasao R., Chaimongkol N., Kongsricharoern T., Ukarapol N., Maneekarn N. (2015). Saffold viruses in pediatric patients with diarrhea in Thailand. J. Med. Virol..

[6] Zoll J., Erkens Hulshof S., Lanke K., Verduyn Lunel F., Melchers W.J., Schoondermark-van de Ven E., Roivainen M., Galama J.M., van Kuppeveld F.J. (2009). Saffold virus, a human theiler’s-like cardiovirus, is ubiquitous and causes infection early in life. PLoS Pathog..

[7] Abed Y., Boivin G. (2008). New saffold cardioviruses in 3 children, Canada. Emerg. Infect. Dis..

[8] Drexler J.F., Luna L.K., Stocker A., Almeida P.S., Ribeiro T.C., Petersen N., Herzog P., Pedroso C., Huppertz H.I., Ribeiro Hda C. (2008). Circulation of 3 lineages of a novel saffold cardiovirus in humans. Emerg. Infect. Dis..

[9] Nix W.A., Khetsuriani N., Penaranda S., Maher K., Venczel L., Cselko Z., Freire M.C., Cisterna D., Lema C.L., Rosales P. (2013). Diversity of picornaviruses in rural bolivia. J. Gen. Virol..

[10] Lamson D., Renwick N., Kapoor V., Liu Z., Palacios G., Ju J., Dean A., St George K., Briese T., Lipkin W.I. (2006). Masstag polymerase-chain-reaction detection of respiratory pathogens, including a new rhinovirus genotype, that caused influenza-like illness in New York state during 2004–2005. J. Infect. Dis..

[11] Dominguez S.R., Briese T., Palacios G., Hui J., Villari J., Kapoor V., Tokarz R., Glode M.P., Anderson M.S., Robinson C.C. (2008). Multiplex MassTag-PCR for respiratory pathogens in pediatric nasopharyngeal washes negative by conventional diagnostic testing shows a high prevalence of viruses belonging to a newly recognized rhinovirus clade. J. Clin. Virol..

[12] Djikeng A., Halpin R., Kuzmickas R., Depasse J., Feldblyum J., Sengamalay N., Afonso C., Zhang X., Anderson N.G., Ghedin E. (2008). Viral genome sequencing by random priming methods. BMC Genomics.

[13] Martin M. (2011). Cutadapt removes adapter sequences from high-throughput sequencing reads. EMBnet.journal.

[14] Schmieder R., Edwards R. (2011). Quality control and preprocessing of metagenomic datasets. Bioinformatics.

[15] Boisvert S., Raymond F., Godzaridis E., Laviolette F., Corbeil J. (2012). Ray meta: Scalable de novo metagenome assembly and profiling. Genome Biol..

[16] Edgar R.C. (2004). Muscle: Multiple sequence alignment with high accuracy and high throughput. Nucleic Acids Res..

[17] Tamura K., Stecher G., Peterson D., Filipski A., Kumar S. (2013). Mega6: Molecular evolutionary genetics analysis version 6.0. Mol. Biol. Evol..

[18] Czechowicz J., Huaman J.L., Forshey B.M., Morrison A.C., Castillo R., Huaman A., Caceda R., Eza D., Rocha C., Blair P.J. (2011). Prevalence and risk factors for encephalomyocarditis virus infection in Peru. Vector Borne Zoonotic Dis..

[19] Oberste M.S., Gotuzzo E., Blair P., Nix W.A., Ksiazek T.G., Comer J.A., Rollin P., Goldsmith C.S., Olson J., Kochel T.J. (2009). Human febrile illness caused by encephalomyocarditis virus infection, Peru. Emerg. Infect. Dis..

