# Supplementary material for: Full Genomic Characterization of a Saffold Virus Isolated in Peru
Source: Pathogens. 2015 Nov 20;4(4):816–25. doi: 10.3390/pathogens4040816 (PMC4693166; doi:10.3390/pathogens4040816)
Supplement: Supplementary File 1 [file pathogens-04-00816-s001.pdf]

*Pathogens* **2015**, *4*, 816-825; doi:10.3390/pathogens4040816

OPEN ACCESS

*pathogens*

**ISSN 2076-0817**

www.mdpi.com/journal/pathogens

*Supplementary Materials*

# Full Genomic Characterization of a Saffold Virus Isolated in Peru

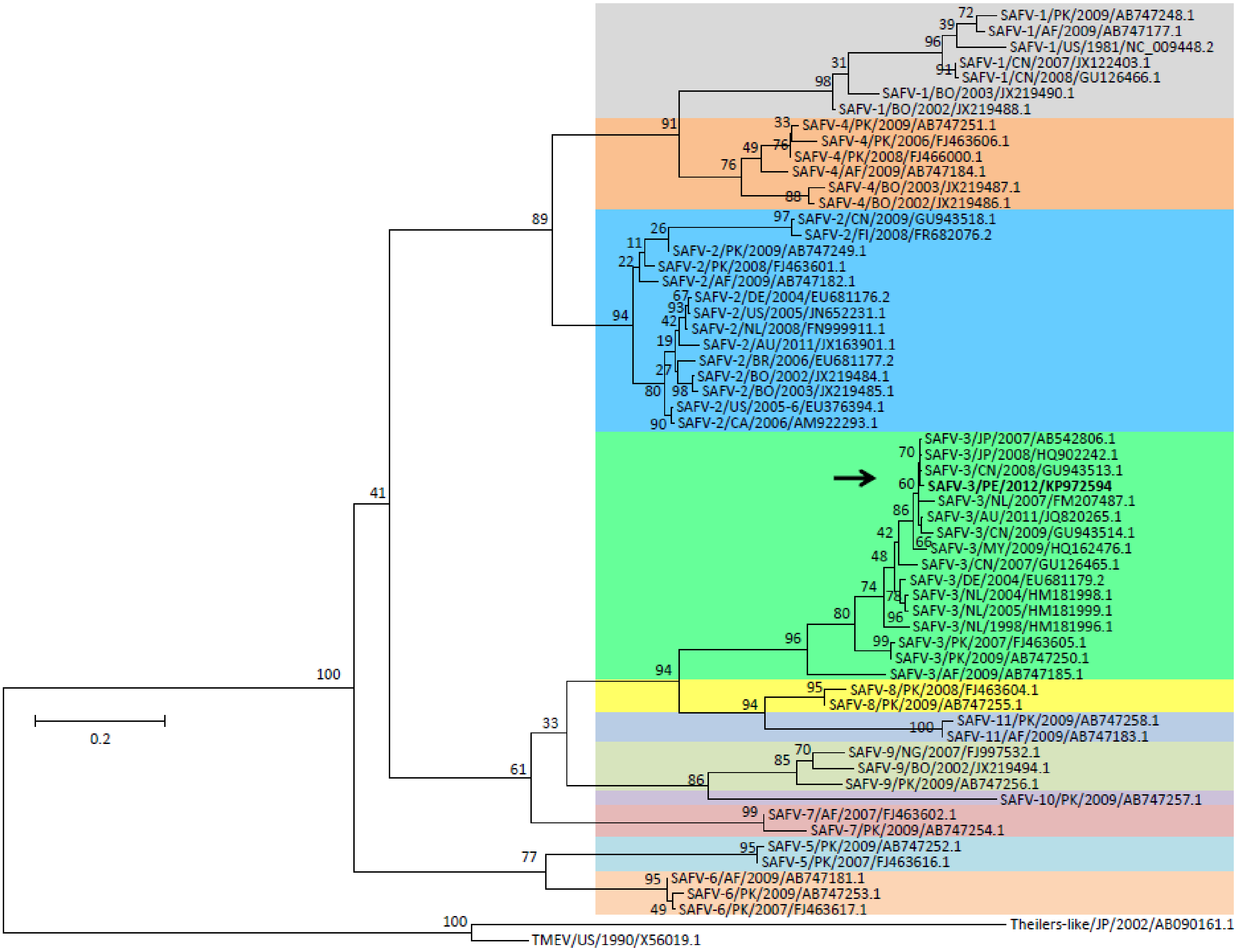


**Figure S1.** Phylogenetic analysis of SAFV partial VP1 (B) sequences. Each strain is labeled using standard identifiers, including virus type, country of isolation (using ISO country codes), isolate name (if any), year of isolation and GenBank accession number. Additionally, SAFV1-11 serotypes are color coded for easy viewing. The Peruvian isolate described here is highlighted in bold and with an arrow. The tree was constructed using a total of 53 publicly available complete VP1 sequences covering serotypes 1–11 plus 7 additional partial VP1 sequences. Scale bars represent the number of substitutions per site.

© 2015 by the authors; licensee MDPI, Basel, Switzerland. This article is an open access article distributed under the terms and conditions of the Creative Commons Attribution license (http://creativecommons.org/licenses/by/4.0/).
